# Is Characteristic Frequency Limiting Real-Time Electrocochleography During Cochlear Implantation?

**DOI:** 10.3389/fnins.2022.915302

**Published:** 2022-07-22

**Authors:** Amit Walia, Matthew A. Shew, Shannon M. Lefler, Dorina Kallogjeri, Cameron C. Wick, Timothy A. Holden, Nedim Durakovic, Amanda J. Ortmann, Jacques A. Herzog, Craig A. Buchman

**Affiliations:** Department of Otolaryngology—Head and Neck Surgery, Washington University School of Medicine in St. Louis, St. Louis, MO, United States

**Keywords:** cochlear implantation, electrocochleography, hearing preservation, multifrequency electrocochleography, characteristic frequency, 250 Hz vs. 500 Hz, acoustic stimulus

## Abstract

**Objectives:**

Electrocochleography (ECochG) recordings during cochlear implantation have shown promise in estimating the impact on residual hearing. The purpose of the study was (1) to determine whether a 250-Hz stimulus is superior to 500-Hz in detecting residual hearing decrement and if so; (2) to evaluate whether crossing the 500-Hz tonotopic, characteristic frequency (CF) place partly explains the problems experienced using 500-Hz.

**Design:**

Multifrequency ECochG comprising an alternating, interleaved acoustic complex of 250- and 500-Hz stimuli was used to elicit cochlear microphonics (CMs) during insertion. The largest ECochG drops (≥30% reduction in CM) were identified. After insertion, ECochG responses were measured using the individual electrodes along the array for both 250- and 500-Hz stimuli. Univariate regression was used to predict whether 250- or 500-Hz CM drops explained low-frequency pure tone average (LFPTA; 125-, 250-, and 500-Hz) shift at 1-month post-activation. Postoperative CT scans were performed to evaluate cochlear size and angular insertion depth.

**Results:**

For perimodiolar insertions (*N* = 34), there was a stronger linear correlation between the largest ECochG drop using 250-Hz stimulus and LFPTA shift (*r* = 0.58), compared to 500-Hz (*r* = 0.31). The 250- and 500-Hz CM insertion tracings showed an amplitude peak at two different locations, with the 500-Hz peak occurring earlier in most cases than the 250-Hz peak, consistent with tonotopicity. When using the entire array for recordings after insertion, a maximum 500-Hz response was observed 2–6 electrodes basal to the most-apical electrode in 20 cases (58.9%). For insertions where the apical insertion angle is >350 degrees and the cochlear diameter is <9.5 mm, the maximum 500-Hz ECochG response may occur at the non-apical most electrode. For lateral wall insertions (*N* = 14), the maximum 250- and 500-Hz CM response occurred at the most-apical electrode in all but one case.

**Conclusion:**

Using 250-Hz stimulus for ECochG feedback during implantation is more predictive of hearing preservation than 500-Hz. This is due to the electrode passing the 500-Hz CF during insertion which may be misidentified as intracochlear trauma; this is particularly important in subjects with smaller cochlear diameters and deeper insertions. Multifrequency ECochG can be used to differentiate between trauma and advancement of the apical electrode beyond the CF.

## Introduction

Cochlear implantation is the standard of care for the management of moderate-to-profound sensorineural hearing loss in patients who no longer gain benefit from traditional amplification (Carlson, 2020). Over the last two decades, the indications for cochlear implants (CIs) are expanding beyond individuals with profound hearing loss. There is now a greater emphasis on patients with residual low-frequency hearing with poor speech understanding, as CIs have shown to provide significant benefit in both quiet and background noise. Nevertheless, surgeons and audiologists are hesitant to implant patients with functional hearing as implantation can lead to trauma and ultimately loss of residual hearing (Gstoettner et al., 2004; Jia et al., 2013). A major limitation of hearing through a CI is that low-frequency, harmonic pitch information is not represented well by the electrical signal (Gantz and Turner, 2004; Gfeller et al., 2006; Golub et al., 2012; Roland et al., 2016). Thus, CI recipients without residual hearing often struggle with speech perception in background noise and differences in music perception (Carroll et al., 2011). A potential solution is to combine low-frequency acoustic hearing with the electrical hearing from the CI (i.e., electroacoustic stimulation) which leads to improved pitch perception and performance in background noise (Tejani and Brown, 2020).

There are various approaches to maximize hearing preservation using different electrode types, soft surgical techniques, tailoring insertion depths to a patient’s cochlear anatomy, and the application of perioperative steroids; however, there is no clear consensus within the literature on how to reliably preserve residual hearing (Yukawa et al., 2004; Roland et al., 2005; Thong et al., 2017; Lenarz et al., 2019; Snels et al., 2019; Shew et al., 2021). Since surgeons receive little active feedback during insertion of the electrode array, most insertions are performed blindly and likely contribute to the highly variable hearing preservation rates (Gstoettner et al., 2008; Pillsbury et al., 2018).

Recently, electrocochleography (ECochG) has emerged as a tool to provide the surgeon with real-time feedback about potential intracochlear trauma during electrode insertion. Intraoperative, real-time ECochG is now possible, using the implant array itself, to monitor physiologic responses from the cochlear-neural substrate during array insertion with the goal of minimizing intracochlear trauma and maximizing depth of insertion (Dalbert et al., 2015; Adunka et al., 2016; Campbell et al., 2016; Abbas et al., 2017; Harris et al., 2017a,b; Kim et al., 2017; Dalbert et al., 2018; Koka et al., 2018; Fontenot et al., 2019; Giardina et al., 2019; Lenarz et al., 2020; O’Leary et al., 2020). The ongoing ECochG response is composed of the cochlear microphonic (CM), auditory nerve neurophonic (ANN), summating potential, and compound action potential. The CM represents the electric current through the stereocilia of outer hair cells and can be measured using the electrode array. The change in CM has been shown to correlate with the degree and location of trauma in some studies (Campbell et al., 2015; Dalbert et al., 2018; Koka et al., 2018). The hypothesis underlying the body of work focused on using ECochG as an intraoperative tool is that sudden, acute reduction or drops in the ECochG response amplitude during array insertion will indicate electrode-basilar membrane contact and subsequent impaired residual hearing. Few studies have shown that the number and magnitude of amplitude drops correlate with subsequent loss of residual hearing (Campbell et al., 2016; O’Leary et al., 2020).

In most studies using ECochG during electrode insertion, a 500-Hz acoustic pure-tone stimulus is used to measure the physiologic response from the most-apical electrode contact on the implant array. Generally, as the array approaches the apex of the cochlea, the CM amplitude gradually increases since the electrode is theorized to approach the characteristic frequency (CF) place for 500-Hz. If there is a drop in CM amplitude during the insertion, this may be related to (1) electrode-basilar membrane contact resulting in reversible or irreversible intracochlear trauma, (2) advancement of the most-apical electrode beyond the CF place in the cochlea, or (3) exceeding the intracochlear source generator with inconsistent hair cells throughout the cochlea (Campbell et al., 2017; Saoji et al., 2019; Soulby et al., 2021). When a single stimulus frequency of 500-Hz is used for monitoring during the insertion, there is no way of determining which of the above causes resulted in the decreased CM amplitude, which makes it challenging to provide clinically useful feedback intraoperatively. This notion may explain some of the variability across studies using intraoperative real-time ECochG as a hearing preservation tool (Dalbert et al., 2015; Adunka et al., 2016; Campbell et al., 2016; Abbas et al., 2017; Harris et al., 2017a,b; Kim et al., 2017; Dalbert et al., 2018; Koka et al., 2018; Fontenot et al., 2019; Giardina et al., 2019; Lenarz et al., 2020; O’Leary et al., 2020).

Multifrequency ECochG is a technology that has been available recently that allows for simultaneous or alternating stimulus presentation of multiple frequencies for response measurement throughout electrode insertion. Prior studies have exclusively focused on using a single frequency during the insertion with 500-Hz being the most common. To our knowledge, no prior studies have evaluated multifrequency ECochG as a hearing preservation tool, and only one case report (Saoji et al., 2019) to date has assessed the feasibility of intraoperative multifrequency ECochG.

The purpose of the present study was (1) to determine whether a 250-Hz stimulus is superior to 500-Hz in detecting residual hearing decrement and if so; (2) to evaluate whether crossing the 500-Hz tonotopic, CF place partly explains the problems experienced using 500-Hz. Here, an alternating, interleaved acoustic complex of 250- and 500-Hz was used to elicit primarily CMs at different locations along the tonotopic axis of the basilar membrane. Using the most-apical electrode for the recording, changes in CM amplitude were monitored as the electrode array was advanced without any real-time feedback used to optimize the placement of the array.

## Materials and Methods

### Study Population and Inclusion Criteria

A total of 48 adult CI candidates were enrolled in this prospective cohort study and were implanted with either a perimodiolar (CI612 or CI632; Cochlear Corp., Sydney, NSW, Australia) or slim lateral wall electrode array (CI624). Five experienced CI surgeons at a single institution participated in this study. The study was approved by the institutional review board at Washington University in St. Louis (IRB #202007087). All patients provided verbal and written informed consent prior to participation. Eligible patients were adults with preoperative low-frequency pure tone average (LFPTA; 125, 250, and 500-Hz) ≤ 60-dB HL. Patients undergoing revision surgery or those with middle ear pathology were excluded. Additionally, patients without a patent external auditory canal were excluded as the acoustic stimulus is delivered *via* air conduction. Demographic information is provided in Table 1.

**TABLE 1:** Demographic, audiologic, and 3-D CT reconstruction data for all 48 patients who met inclusion criteria and underwent cochlear implantation with multifrequency ECochG (250- and 500-Hz acoustic stimulus).

Electrode array type		Perimodiolar	Lateral wall
Laterality	*Right*	18 (52.9)	7 (50.0)
	*Left*	16 (47.1)	7 (50.0)
Age (years)		68.1 ± 15.9	68.5 ± 13.7
Etiology	*Idiopathic*	19 (55.9)	7 (50.0)
	*Presbycusis*	0	2 (14.3)
	*Noise Induced*	6 (17.6)	2 (14.3)
	*Sudden Sensorineural*	1 (2.9)	2 (14.3)
	*Meniere’s*	1 (2.9)	0
	*Autoimmune*	2 (5.9)	0
	*Congenital*	4 (11.9)	1 (7.1)
	*Infectious*	0	0
	*Trauma*	1 (2.9)	0
Duration of Hearing Loss (years)		24.4 ± 18.3	23.6 ± 16.2
Duration of Severe-to-Profound Hearing Loss (years)		7.6 ± 12.3	4.6 ± 4.3
** Audiologic Thresholds**			
Preoperative Low-Frequency Pure Tone Average (LFPTA; 125, 250, and 500-Hz; dB HL)		59.4 ± 22.8	48.1 ± 11.4
Postoperative LFPTA (dB HL)		92.9 ± 23.7	99.1 ± 20.1
LFPTA Shift (dB HL)		33.6 ± 22.7	51.0 ± 19.4
** CT 3-D Reconstructions**			
Basal Electrode Insertion Angle (deg)		19.7 ± 13.0	0.8 ± 5.2
Apical Electrode Insertion Angle (deg)		396.5 ± 40.2	294.1 ± 41.9
1st Turn Outer Wall Length (mm)		22.5 ± 1.2	23.2 ± 1.3
Cochlear Diameter Center to Round Window (mm)		9.2 ± 0.4	9.4 ± 0.6
Cochlear Diameter Orthogonal to Round Window		7.1 ± 0.4	13.2 ± 0.4
			

### Surgical and Multifrequency Electrocochleography Technique

During insertion, ECochG potentials were measured from the most-apical electrode of the CI array itself using the Cochlear Research Platform Ver 1.2. An ER3-14A insert earphone (Etymotic, Elk Grove Village, IL, United States) was placed into the external auditory canal prior to surgical site sterile preparation. A standard surgical approach was used for all CIs. Once there was adequate exposure of the round window, the CI was seated under the temporalis muscle. The telemetry coil was then placed over the skin in alignment with the CI antennae *via* a sterile ultrasound drape. The most-apical electrode (e22) was first inserted into the round window opening and was conditioned in perilymph with reference to the case ground. During electrode insertion, the auditory stimulus, delivered *via* the insert earphone, consisted of sequential, alternating phase presentations of 250- and 500-Hz tone bursts, with a single repetition per phase. Each tone burst had a duration of 10 ms with a 1-ms onset/offset ramp time. The recording epoch for the insertion recording was 14 ms, starting 1 ms before stimulus onset, with a sampling rate of 20 kHz. The 250- and 500-Hz stimuli were delivered at 108- and 99.5-dB SPL, respectively, which was based on the maximum output of the speaker. Insertion feedback related to the ECochG recordings was not provided to the implanting surgeon, and the electrode was fully inserted according to the manufacturer’s specifications. The ECochG responses were measured using the most-apical electrode (e22) during the insertion.

An electrode recording sweep was performed after the electrode was fully inserted. Responses were recorded across all even electrodes, which resulted in recordings from a total of 11 electrodes. Tone burst stimuli of 250- and 500-Hz stimuli were independently delivered per electrode sweep in condensation and rarefaction starting phases, with 30 repetitions per phase. The stimulus duration was 14 ms with rise and fall times of 1 ms, shaped by a Blackman window. The recording epoch was 18 ms, starting 1 ms before stimulus onset, with a sampling rate of 20 kHz. The intensity of the stimulus was identical to that used for the insertion.

### Electrocochleography Signal Analysis

Electrocochleography responses were processed offline and stored as condensation and rarefaction phases. The difference curve was calculated by subtracting responses to rarefaction from condensation phase stimuli using MATLAB R2020a (MathWorks Corp., Natick, MA, United States) with custom software procedures. From the difference curve, the ongoing portion was selected for faster Fourier transformation (FFT) and the amplitude of the response to 250- and 500-Hz stimulus frequency was determined. The amplitude from the FFT was used to generate the insertion trajectory ECochG response. The FFT was also calculated using the same methods for the electrode sweep ECochG responses. As described in previous studies (Fitzpatrick et al., 2014; McClellan et al., 2014; Fontenot et al., 2019), a significant response was defined as one whose magnitude exceeded the noise floor by 3 standard deviations. The noise floor was ∼1 μV for the insertion (single sweep) and ∼0.3 μV for the electrode sweep (30 sweeps) measurements.

### Imaging, Cochlear Size, and Insertion Depth

**FIGURE 1:**
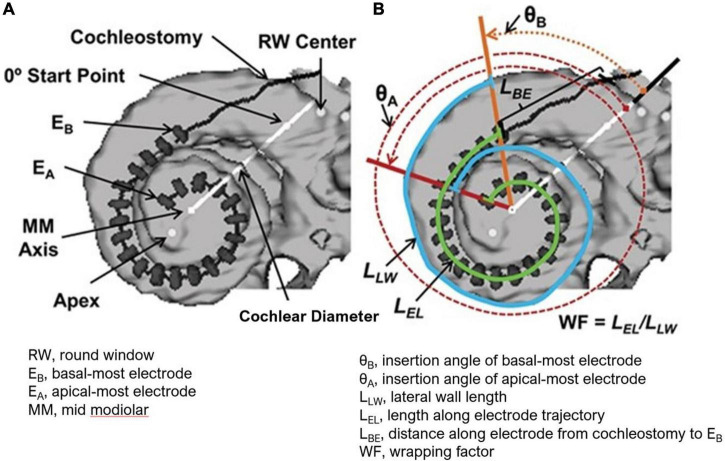
**(A)** 3-D Reconstruction from CT of a CI recipient’s electrode array with dark line and gray hash markers used to measure all 22 electrode positions. The cochlear diameter as shown is measured from the center of the round window through the mid-modiolar axis to the lateral wall. **(B)** Measurements used to obtain array insertion depth and the mediolateral position of the array (wrapping factors, WF) are shown. These include the lateral wall length (*L*_LW_) from the angular position of E_B_ to the angular position of E_A_ (blue line), the length along the electrode trajectory (*L*_EL_) from E_B_ to E_A_ (green line), and the distance along the electrode array from the cochleostomy to E_B_ (*L*_BE_). The electrode insertion length to E_A_ is then the sum of lengths *L*_BE_ and *L*_EL_. Figure reprinted from Holden et al. (2013) with permission from Wolters Kluwer Health, Inc. The Creative Commons license does not apply to this content. Use of the material in any format is prohibited without written permission from the publisher, Wolters Kluwer Health, Inc. Please contact permissions@lww.com for further information.

The electrode position and cochlear anatomic properties were further interrogated in select patients (*N* = 33) who were willing to undergo postoperative computed tomography (CT) scans and 3D reconstructions as previously reported by Skinner et al. (2007) and Teymouri et al. (2011) (Figure 1). The angular insertion depth, cochlear diameter, and number of electrodes within each scala were determined. The cochlear diameter is measured from the center of the round window through the mid-modiolar axis to the lateral wall. The measurement is dependent on viewing the cochlea in the mid-modiolar axis centered volume and defining the cochlear canal wall using a set Hounsfield value.

### Audiometric Thresholds

Pre- and postoperative audiograms were administered to document the impact of cochlear implantation on native acoustic hearing at 1-month post-activation. The audiogram closest to the time of implantation was considered the preoperative audiogram. Although tympanograms were not done before audiometric measurements in subjects postoperatively to ensure there was no conductive loss due to fluid after surgery, the middle ear status was verified by otoscopy. Low-frequency pure tone average (LFPTA; 125, 250, and 500-Hz) shift from preoperative to 1-month post-activation as measured from the audiogram was the primary outcome evaluated.

### Statistical Analysis

The primary objective of this study was to compare the stability of CM amplitude for 250- and 500-Hz during insertion with the stability of the postoperative audiogram following surgery. Specifically, the drops in CM amplitude (FFT of the difference curve) during the insertion for 250- and 500-Hz, independently, were compared with the shift in the LFPTA from preoperative to postoperative behavioral audiogram testing at 1-month post-activation. A CM amplitude drop was considered significant when a change of at least 30% of a prior maximum amplitude was observed during insertion. The CM amplitude drop was calculated as the change in amplitude from the peak (maximum amplitude response) to the largest drop point after the peak, during the insertion recordings. ECochG responses were measured in μVs and subsequently converted to a logarithmic scale (dB relative to 1 μV). Normality was confirmed using the Shapiro-Wilk test. Thus, the Pearson correlation (*r*) was used to determine the strength of relationships between CM amplitude drop for 250- and 500-Hz independently, and LFPTA shift. In addition to CM amplitude drop, the starting CM amplitude, number of CM amplitude drops, final CM amplitude compared to maximum CM response during insertion, type of insertion pattern, electrode type, and insertion time were compared with LFPTA shift using univariate linear regression. The insertion patterns were previously defined in Harris et al. (2017a) – (1) Type A, overall increase in amplitude from the beginning of insertion until completion; (2) Type B, maximum amplitude at start of insertion with decrease in amplitude as the electrode is further inserted; (3) Type C, similar amplitude at start and end of insertion with maximum amplitude at the middle of insertion. For the lateral wall electrode, non-parametric testing including Spearman correlation (ρ) was used as the ECochG responses and audiogram thresholds were not normally distributed, likely due to limited sample size.

The secondary objective of this study was to understand whether the tonotopic, CF place for 500-Hz affected the ability to use the 500-Hz acoustic stimulus during electrode insertion. Univariate linear regression was used to assess whether cochlear diameter and angular insertion depth were able to predict whether the 500-Hz maximum ECochG response along the electrode array after insertion was located basal to the apical-most electrode (e22), suggesting that e22 had crossed the 500-Hz CF place. Analyses were performed with SPSS 27 for Windows (IBM Corp., Armonk, NY, United States). Alpha levels for all statistical tests were set at 0.05 and were two-tailed.

## Results

### Participant Demographics and Characteristics

**FIGURE 2:**
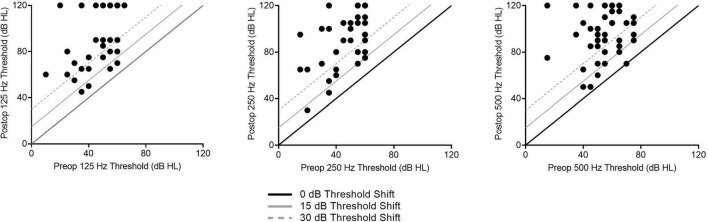
Individual low-frequency hearing preservation results as related to audiogram thresholds shifts for 1-month post-activation are shown here for all subjects.

Of the 48 patients included within the study, the average age was 67.8 ± 15.7 years at the time of implantation. Thirty-four subjects received the perimodiolar electrode (70.8%) and 14 subjects received the slim lateral wall electrode (29.2%). The average LFPTA preoperatively was 56.1 ± 20.7 dB HL and postoperatively at 1-month post-activation was 90.4 ± 25.8 dB HL, with an LFPTA shift of 35.7 ± 24.1 dB HL. Threshold shifts across individual frequencies at 1-month post-activation are shown in Figure 2. Further demographic information can be found in Table 1.

### Multifrequency Electrocochleography Recordings During Insertion

**FIGURE 3:**
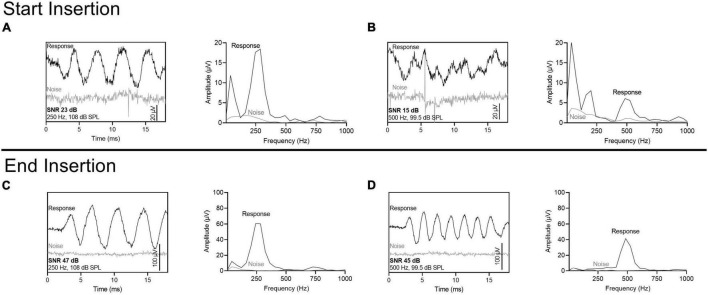
Patient examples of ECochG response to multifrequency ECochG using 250- and 500-Hz tone burst. **(A–D)** Show four different insertion tracks. **(A)** No asynchrony between 250- and 500-Hz response. **(B)** Overall rise in response with one dyssynchronous point (marked with black arrow) where there is stability in 250-Hz response and drop in 500-Hz response. **(C,D)** Multiple dyssynchronous points (black arrows) noted between 250- and 500-Hz response with overall rise in 250-Hz response and drop in 500-Hz response. CM Drops >30% are shown as asterisks.

**FIGURE 4:**
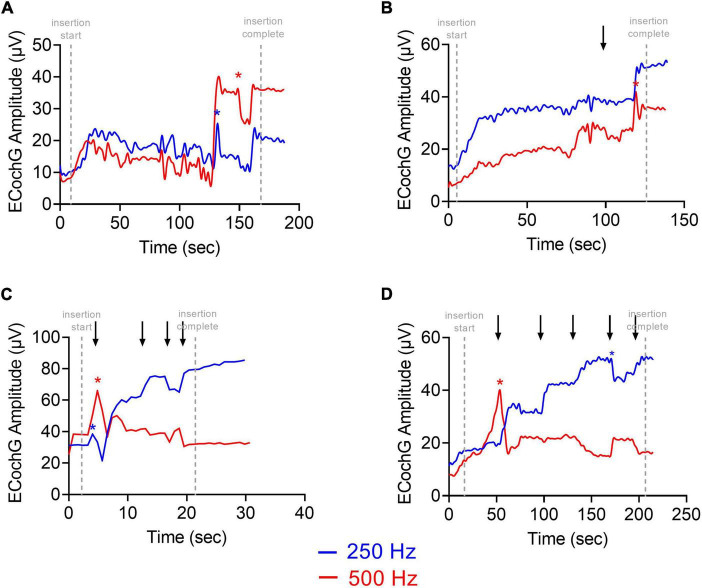
ECochG recording (difference curves) using the most apical electrode at the start of the insertion and at the end of insertion. Stimulus parameters were sequential, alternating phase presentations of 250- and 500-Hz tone bursts, with a single repetition per phase. Fast Fourier transformations are also shown. The noise floor was measured with the sound tube clamped. The signal-to-noise ratio (SNR) for 250-Hz was larger than 500-Hz at the start of the insertion but was similar at the end of insertion. **(A)** 250-Hz stimulus at the start of insertion. **(B)** 500-Hz stimulus at the start of insertion. **(C)** 250-Hz stimulus at the end of insertion. **(D)** 500-Hz stimulus at the end of insertion.

Four ECochG insertion tracks are plotted in Figure 3. Panel A shows a representative example using alternating, interleaved 250- and 500-Hz acoustic stimuli, where there are highly correlated rises and drops in CM amplitude throughout the insertion between the two frequencies. The ECochG insertion trajectories in B, C, and D show some discordance (demarcated with black arrows) between the 250- and 500-Hz CM amplitude responses. There are different points during the insertion where there is a rise in the 250-Hz CM amplitude and a drop in the 500-Hz CM amplitude and vice versa. This asynchrony between the two responses was the primary motivation of this study. The difference curves of an example recording, both at the start and end of insertion, are shown in Figure 4 with the FFT for both 250- and 500-Hz stimuli.

### Comparing 250- and 500-Hz Cochlear Microphonic Drops With Hearing Preservation for Perimodiolar Electrode

**FIGURE 5:**
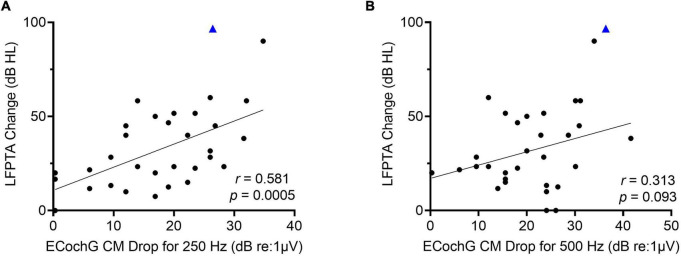
**(A)** There is a moderately strong linear correlation between low-frequency pure tone average (LFPTA; 125, 250, and 500-Hz) shift from preoperative to 1-month post-activation and largest ECochG amplitude drop for 250-Hz. **(B)** There is no linear correlation between LFPTA change and largest ECochG amplitude drop for 500-Hz. Outlier is demarcated as blue triangle, which required two separate insertions as first insertion resulted in tip rollover.

Of the 34 participants who received the perimodiolar electrode, univariate linear regression was used to explore the association of LFPTA shift with ECochG insertion trajectory characteristics (independently, for 250- and 500-Hz) including starting CM amplitude, final CM amplitude compared to maximum CM during insertion, and type of insertion pattern; none of these variables were significant predictors of LFPTA shift (Table 2). A paired *t*-test showed no difference between preoperative audiogram thresholds at 250 and 500-Hz (*p* = 0.312). The only two variables that significantly correlated with LFPTA shift were number of CM amplitude drops for 250-Hz (Pearson Correlation coefficient *r* = 0.39, *p* = 0.018) and amplitude of the largest CM drop for 250-Hz (*r* = 0.58, *p* = 0.0005) as shown in Figure 5. The amplitude of the largest CM drop for 500-Hz was not significantly correlated with LFPTA shift (*r* = 0.31, *p* = 0.093). We compared the two correlations by conducting a Pearson and Filon’s *z* test using the package *cocor* (Diedenhofen and Diedenhofen, 2016). Results indicated that indeed the correlation between the 250-Hz CM drop and LFPTA change was significantly better (*z* = 2.31, *p* = 0.01). The linear fit estimated a y-intercept of 7.1- and 6.8-dB HL for 250- and 500-Hz CM drop, respectively, which represents the estimated LFPTA change observed independent of ECochG CM drops (i.e., no change in ECochG predicted to result in ∼7.0 dB HL LFPTA shift on average but with a wide variance of 2.1–30.1 for 250-Hz ECochG CM drop and 12.7–40.6 for 500-Hz ECochG CM drop). The slope was 3.7- and 3.3-dB HL LFPTA change per 10 dB ECochG CM drop for 250- and 500-Hz, respectively. There was one outlier demarcated as a blue triangle in Figure 5, which required two separate insertions as first insertion resulted in a tip rollover. Sensitivity analysis performed with this case excluded from the analysis showed no change in study findings.

**TABLE 2:** Comparison of multifrequency electrocochleography insertion trajectory parameters using 250- and 500-Hz acoustic stimulus and univariate linear regression with low-frequency pure tone average (125, 250, and 500-Hz) threshold shift from preoperative to 1-month post-activation for all insertions (both perimodiolar and lateral wall electrodes).

		250-Hz			500-Hz		
		Mean ± STD or N (%)	*r*	*p*	Mean ± STD or N (%)	*r*	*p*
Number of Cochlear Microphonic (CM) Amplitude Drops (>2 μV)		2.2 ± 2.3	0.39	0.018	2.1 ± 1.4	0.34	0.05
Largest CM Drop (dB re: 1 μV)		2.3 ± 3.7	0.52	0.002	9.3 ± 6.0	0.39	0.033
Starting CM Amplitude (μV)		11.7 ± 11.7	0.161	0.359	9.1 ± 14.9	0.266	0.117
Final CM Amplitude/Max CM During Insertion * 100%		81.0 ± 20.6	0.023	0.897	70.0 ± 26.5	0.092	0.593
Type of Insertion Pattern	*Type A*	26 (76.5)	0.112	0.529	11 (32.4)	0.207	0.225
	*Type B*	6 (17.6)			10 (29.4)		
	*Type C*	2 (5.9)			13 (38.2)		

**FIGURE 6:**
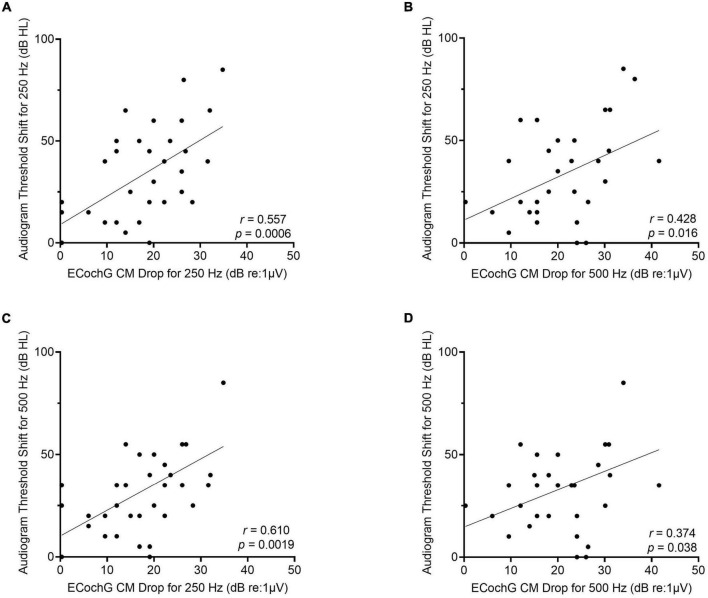
For the perimodiolar electrode, changes in ECochG CM drops for 250-Hz are more strongly correlated with frequency-specific audiogram threshold shift at both 250- and 500-Hz compared to ECochG CM drops when using 500-Hz stimulus. **(A)** Moderately strong linear correlation between audiogram threshold shift at 250-Hz from preoperative to 1-month post-activation and largest ECochG CM drop for 250-Hz. **(B)** Moderately strong linear correlation between audiogram threshold shift at 250-Hz and largest ECochG CM drop for 500-Hz. **(C)** Moderately strong linear correlation between audiogram threshold shift at 500-Hz and largest ECochG CM drop for 250-Hz. **(D)** Weak linear correlation between audiogram threshold shift at 500-Hz and largest ECochG CM drop for 500-Hz.

The frequency-specific threshold shift on the behavioral audiogram from preoperative to 1-month post-activation was then compared to the ECochG CM drop for 250- and 500-Hz. There was a moderately strong linear correlation between ECochG CM drop using 250-Hz acoustic stimulus and audiogram threshold shift for 250-Hz (*r* = 0.56, *p* = 0.0006; Figure 6). Similarly, there was a moderately strong linear correlation between ECochG CM drop for 250-Hz and audiogram threshold shift for 500-Hz (*r* = 0.61, *p* = 0.002). Although ECochG CM drop for 500-Hz was moderately correlated with audiogram threshold shifts for both 250-Hz (*r* = 0.43, *p* = 0.02) and 500-Hz (*r* = 0.37, *p* = 0.04), ECochG CM changes using 250-Hz stimulus were more strongly correlated with both 250- and 500-Hz frequency-specific shifts on audiogram than ECochG CM changes using 500-Hz stimulus.

### Relationship of Cochlear Size to Angular Insertion Depth

**FIGURE 7:**
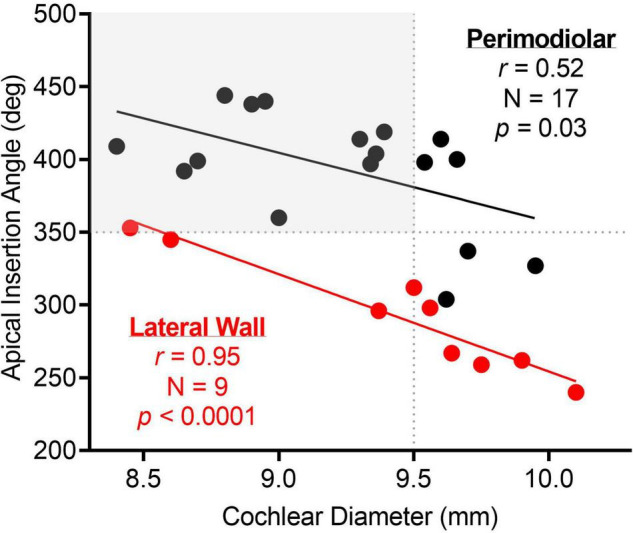
There was a strong linear correlation between apical insertion angle and cochlear diameter for both perimodiolar and lateral wall electrodes. The dotted lines highlight the apical insertion angle and cochlear diameter where the electrode sweep results in a maximum amplitude CM at a more basal electrode than the most-apical one for 500-Hz stimulus frequency. As highlighted in gray, a cochlear diameter less than 9.5 mm and an apical insertion angle greater than 350 degrees may result in the most-apical electrode crossing the CF place for 500-Hz.

The mean cochlear diameter was 9.3 ± 0.5 mm (range, 8.4–10.1), and the mean angular insertion depth was 358.8 ± 62.7 degrees (range, 240.0–444.0) as measured from 3-D reconstruction of CT temporal bone imaging. Figure 7 shows there was a strong linear correlation between cochlear diameter and angular insertion depth for both the perimodiolar (*r* = 0.52, *p* = 0.03) and lateral wall (*r* = 0.95, *p* < 0.0001) arrays, where the angular insertion depth tended to decrease as the cochlear diameter increased.

### Electrocochleography Electrode Sweep and Insertion Pattern for Perimodiolar Electrodes

**FIGURE 8:**
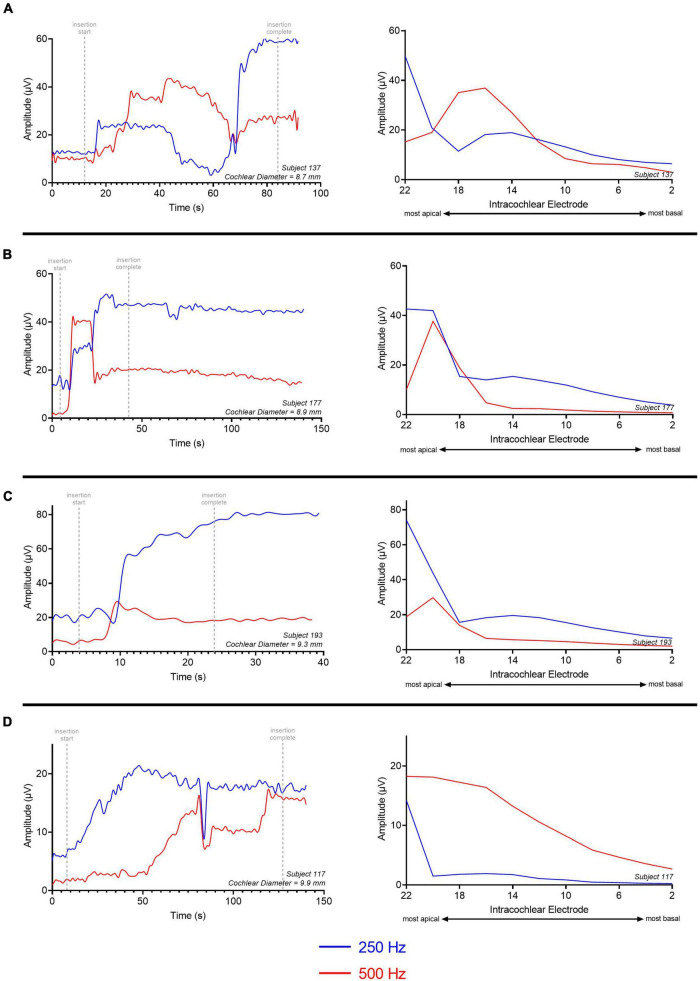
Multifrequency ECochG with 250- and 500-Hz acoustic stimulus is measured during the insertion of the electrode array. Responses were measured across the electrode array with independent presentations of 250 (red) and 500-Hz (blue) after full insertion (electrode sweep). The cochlear diameter across these examples increases in length from **(A–D)**. **(A)** There is a gradual rise in ECochG response for both 250- and 500-Hz acoustic stimulus; however, the 500-Hz ECochG response continues to drop during insertion of the last 4–6 electrodes while the 250-Hz ECochG response continues to rise. The electrode sweep shows a maximum ECochG response at electrode 16 and electrode 22 for 500 and 250-Hz, respectively. **(B,C)** Gradual rise in ECochG response for 250- and 500-Hz with a drop in 500-Hz response during insertion of last 2–4 electrodes. Electrode sweep shows a maximum ECochG response at electrode 20 and electrode 22 for 500 and 250-Hz, respectively. **(D)** Gradual rise in ECochG response for both 250- and 500-Hz acoustic stimulus throughout the insertion with congruent drop across 250- and 500-Hz. Electrode sweep shows a maximum ECochG response at electrode 22 for both 250- and 500-Hz acoustic stimulus. This example was a patient with a large cochlea (cochlear diameter from RW to modiolus = 9.9 mm) as measured from 3-D reconstructions of postoperative temporal bone imaging.

Four representative examples of insertions and electrode sweeps are shown in Figure 8 comparing the insertion pattern using the most-apical electrode and the ECochG responses across the array after full insertion for 4 cochleas of increasing size. For cochleas with shorter diameters (Figures 8A–C), there was a drop in ECochG response for 500-Hz at the end of the insertion (last 1–6 electrodes inserted), while there was a continued rise in response for 250-Hz. This was also observed in the ECochG responses across the array after full insertion. By contrast, the cochleas with larger diameters demonstrated rising insertion patterns for both 250- and 500-Hz as there was a rise in response for both frequencies throughout most of the insertion; ECochG responses along the entire array after full insertion also showed a maximum ECochG response at the most-apical electrode using both 250- and 500-Hz stimuli (Figure 8D).

For all subjects who received the perimodiolar electrode array, the location of the intracochlear electrode with the maximum amplitude CM response along the array was determined for the 250- and 500-Hz acoustic stimulus after full insertion. For 41.1% (*N* = 14/34) of the subjects, the maximum ECochG response was present at the most-apical electrode (e22) for both the 250- and 500-Hz acoustic stimulus. The other 20 subjects had the largest ECochG response for 500-Hz at an electrode located basal to e22 (i.e., e12 to e21). In most cases (85.3%, *N* = 29), the maximum ECochG response for 250-Hz was at the most-apical electrode when recording along the entire array after insertion. For the 5 cases where the 250-Hz maximum CM amplitude was not at the most-apical electrode, there was a simultaneous drop in both the 250-Hz and 500-Hz ECochG responses at the end of insertion; this may be reflective of basilar membrane trauma at the end of insertion.

### Correlation of Electrode Sweep With Cochlear Size and Angular Insertion Depth for Perimodiolar Electrode

**FIGURE 9:**
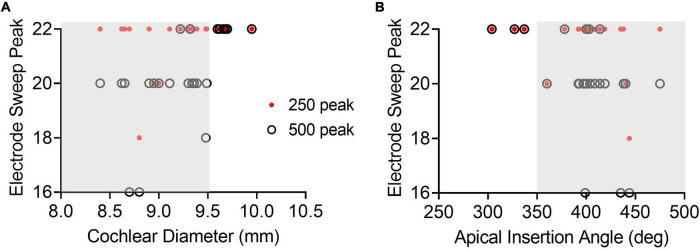
The shorter the cochlear diameter (<9.5 mm) and the deeper the apical insertion angle (>350 degrees), the more likely the 500-Hz maximum on the electrode sweep would present at a more basal intracochlear electrode than the most-apical one (i.e., the more likely the electrode is to cross the CF place for 500-Hz). **(A)** Maximum ECochG response along the entire array for both 250- and 500-Hz (i.e., electrode sweep maximum) as a function of cochlear diameter. The gray region highlights that for cochlear diameter <9.5 mm, where there may be a possibility that the perimodiolar electrode may pass the 500-Hz CF during the insertion. **(B)** Maximum ECochG response on electrode sweep for both 250- and 500-Hz as a function of apical insertion angle. For apical insertion angles >350 degrees, there is a possibility that the electrode may pass the CF for 500-Hz during the insertion. Electrode sweep peak is numbered from 1–22, where 22 is the most-apical electrode and 1 is the most basal electrode.

The relationship between angular insertion depth or cochlear diameter and ECochG responses along the entire array after insertion for the perimodiolar electrode array is shown in Figure 9. There was a strong linear correlation between cochlear diameter and the intracochlear electrode with the largest ECochG CM response for 500-Hz (*r* = 0.57, *p* = 0.004). Generally, smaller cochleas had the maximum ECochG CM response for 500-Hz at an intracochlear electrode located basal to e22. Thus, for cochleas with diameter lengths >9.5 mm, the ECochG maximum for 500-Hz stimulation on the electrode sweep was always present at e22. This similar strong linear correlation was also present when comparing apical electrode insertion angle with the maximum ECochG CM response for 500-Hz (*r* = 0.49, *p* = 0.017), where a deeper insertion resulted in the 500-Hz peak on the electrode sweep at a more basal electrode than e22. For insertions with apical insertion angles >350 degrees, the ECochG maximum for 500-Hz on the electrode sweep was at a more basal electrode than e22 in 20 cases (58.9%). There were no significant linear correlations between the peak 250-Hz ECochG response along the entire electrode and the cochlear diameter (*r* = 0.31, *p* = 0.15) and apical insertion angle (*r* = 0.22, *p* = 0.32).

### Multifrequency Electrocochleography for Lateral Wall Electrodes

Similar to the perimodiolar electrode, for all lateral wall electrode insertions, the ECochG responses were recorded off of the most-apical electrode during the insertion using both 250- and 500-Hz acoustic stimuli. For all insertions, there was a gradual rise in both 250- and 500-Hz ECochG CM response with a dyssynchronous ECochG response between the two frequencies during only one insertion. Only largest CM amplitude drop for 250-Hz (Spearman ρ = 0.58, *p* = 0.04) and largest CM amplitude drop for 500-Hz (Spearman ρ = 0.67, *p* = 0.01) correlated with LFPTA shift. Starting CM amplitude, final CM amplitude compared to maximum CM during insertion, and type of insertion pattern did not correlate with LFPTA shift.

The average apical insertion angle for the lateral wall electrode was 293.9 ± 44.8 degrees for an average cochlear diameter of 9.4 ± 0.6 mm (Figure 7). For the lateral wall electrode insertions, the maximum ECochG response when recording along the entire array, after full insertion, for both 250- and 500-Hz acoustic stimuli was at the most-apical electrode (e22). There was one exception where the largest response for 250-Hz was at e22 and the largest response for 500-Hz was at e20. This subject had the greatest angular insertion depth (358 degrees) in a smaller cochlea (cochlear diameter 8.4 mm) compared to the rest of the lateral wall electrode insertions.

## Discussion

This single-institution study was conducted to expand what is known about real-time ECochG amplitude drops and subsequent hearing preservation using multifrequency ECochG for subjects undergoing CI surgery. Multifrequency ECochG is a recently available technology that allows for measurement of alternating frequencies during insertion of the electrode array using the CI itself as the recording electrode. The drop in CM amplitude was our primary outcome when using 250- and 500-Hz acoustic stimulus for the ECochG recording during insertion. We hypothesized that a drop in CM amplitude for 250-Hz was more predictive of hearing preservation than changes in CM amplitude for 500-Hz stimulus.

Several recent studies (Harris et al., 2017a; O’Connell et al., 2017; Ramos-Macias et al., 2019; Bester et al., 2021; Lenarz et al., 2022; Walia et al., 2022) have explored intraoperative CM amplitude drops during insertion and its correlation with postoperative behavioral thresholds with no clear consensus on any reliable or significant correlation. Universally, across all prior studies, 500-Hz was used for the acoustic stimulus throughout the insertion. Although there is no clear rationale in these prior studies as to why 500-Hz was selected for the stimulus frequency, one potential explanation is that a tone burst stimulus of 500-Hz for a duration of 8 ms would produce 4 cycles, which would be sufficiently long to identify the CM during the ongoing ECochG response. A lower frequency tone burst stimulus such as 250-Hz for a duration of 12 ms would only produce 3 cycles, which potentially may not be sufficient to identify any changes in the CM during insertion. No prior study has evaluated a 250-Hz acoustic stimulus used to measure real-time ECochG responses and its relationship with postoperative acoustic hearing loss.

### 250- vs. 500-Hz Stimulus – Real-Time Electrocochleography for Hearing Preservation

Saoji et al. (2019) were the first to report measurement of multifrequency ECochG during electrode insertion in a case report format for a single patient. They found that CM tracings from the most-apical electrode resulted in different frequency-specific instances of amplitude peaks during the insertion, which they suggested was consistent with the tonotopic organization of the cochlea. For this study, we were primarily motivated by the discordance between the 250- and 500-Hz ECochG amplitude responses during the insertion, which made it challenging to provide constructive feedback to the surgeon using the newly available multifrequency ECochG. An important difference between the (Saoji et al., 2019) study and the current one is the stimulus parameters of the multifrequency ECochG—the pure tone stimulus for the current study alternated between 250- and 500-Hz throughout the insertion while the Advanced Bionics Active Insertion Monitoring system presents the stimulus simultaneously across multiple frequencies. The impact of simultaneous vs. alternating presentation of the stimulus using multiple frequencies is unknown and may impact its ability to be used as a hearing preservation tool.

The correlation shown in this study between CM amplitude drop for 500-Hz and hearing preservation as measured by LFPTA shift was similar to that shown by Lenarz et al. (2022) and O’Leary et al. (2020). Lenarz et al. used the Advanced Bionics MidScala and SlimJ electrode arrays and O’Leary et al. used straight array from Cochlear Corp. (CI422 and CI522). They both found that CI recipients with ECochG drops of >30% during the insertion had overall worse hearing preservation when compared to subjects with no ECochG drops. Additionally, based on our experience and that noted within these previous studies, a human observer viewing the live recordings and providing the surgeon with active feedback is mostly able to detect >30% changes in CM. As both studies used ECochG recording equipment from different manufacturers, the results emphasized the generalizability of the findings across the various devices. In both studies, neither 250-Hz acoustic stimulus nor multifrequency ECochG was used during the insertion; both used 500-Hz acoustic stimulus only. Soulby et al. (2021) showed the relationship between changes in the intraoperative ECochG CM 500-Hz response both on the electrode sweep and during insertion correlated with postoperative behavioral audiogram thresholds when using the SlimJ electrode array. This is consistent with the findings here as these short slim lateral wall electrodes do not cross the 500-Hz CF place in most cochleae.

Here, we found a stronger linear correlation between the magnitude of ECochG CM drop for 250-Hz and LFPTA change at 1-month post-activation (*r* = 0.58) compared to the ECochG CM drop for 500-Hz and the LFPTA change at 1-month post-activation (*r* = 0.31). The slope was ∼3 dB HL of hearing loss on LFPTA per 10 dB of ECochG CM drop for 250-Hz. Importantly, the slope here is shallow where a large ECochG drop resulted in a small increment of hearing loss. However, it is clear that the surgical effects on insertion, as measured by ECochG CM drops, have a direct impact on hearing preservation.

Frequency-specific threshold shifts on audiogram were also evaluated for ECochG CM drops for 250 and 500-Hz. ECochG CM drop for 250-Hz was the best at predicting audiogram threshold shift for both 250-Hz (*r* = 0.56) and 500-Hz (*r* = 0.61), while ECochG CM drop for 500-Hz was moderately correlated with audiogram threshold shift for both 250-Hz (*r* = 0.43) and 500-Hz (*r* = 0.37). These data show that larger ECochG CM amplitude drops during array insertion using 250-Hz stimulation portends larger degrees of hearing loss compared to 500-Hz stimulation, as measured by both LFPTA and frequency-specific threshold shifts, at postoperative follow-up audiometric testing. The multifrequency ECochG showing ECochG CM drop for both frequencies will likely provide the best intraoperative feedback to optimize hearing preservation; however, if multifrequency ECochG is unavailable, monitoring and responding to ECochG CM drop for 250-Hz will result in the smallest LFPTA shift and audiogram frequency-specific threshold shift for both 250- and 500-Hz.

Other factors that did not show a relationship with hearing preservation included starting CM amplitude, final CM amplitude compared to maximum CM during insertion, and type of insertion pattern. This was similar to the findings from Lenarz et al. (2022). It is reasonable to expect that final ECochG responses are often not reflective of hearing preservation since the final ECochG response does not reflect what may have happened during the electrode insertion. The ECochG drop and its relationship with hearing preservation were significant where a large drop (≥30% reduction in CM) is likely reflective of insertion trauma that affects postoperative acoustic hearing, unlike the other ECochG measures that are not sensitive indicators to measure insertion trauma.

### Characteristic Frequency Place May Explain Importance of 250-Hz Electrocochleography Monitoring for Hearing Preservation

In this study, CM amplitude peaks for 250- and 500-Hz on the electrode sweep occurred at separate intracochlear locations consistent with the tonotopic arrangement of the cochlea in 58.9% of subjects implanted with the perimodiolar electrode. The other 41.1% of subjects had a maximum CM amplitude peak at the most-apical electrode (i.e., e22) for both 250- and 500-Hz. We found that patients with larger cochleas (cochlear diameter ≥9.5 mm) and shallow insertions (<350 degrees) had the CM amplitude peak for both 250- and 500-Hz at the most-apical electrode. We suspect that the electrode array in these cases, after full insertion, did not cross the CF. This is a critical finding as a drop in the CM amplitude of 500-Hz may not be related to intracochlear trauma, but rather crossing of the CF in a majority of cases, which limits the utility of 500-Hz as the stimulus frequency for monitoring intracochlear trauma. The only cases where CM amplitude peak for 250-Hz was seen at a more basal electrode than the most-apical were ones where the CM amplitude dropped for both 250- and 500-Hz; these cases also had the largest LFPTA shift. Thus, during recording of multifrequency ECochG, a synchronous drop in both 250- and 500-Hz is likely related to intracochlear trauma, while a rise in 250-Hz response and a dyssynchronous simultaneous drop in 500-Hz response may be related to crossing of the CF. It is unlikely that the electrode arrays used within this study crossed the CF for 250-Hz.

Intracochlear recordings using electrode sweep provide a unique opportunity to understand the acoustically evoked electrophysiologic responses of the inner ear. Campbell et al. (2017) used intraoperative intracochlear ECochG recordings to describe the concept of sound inducing a traveling wave that propagates down the basilar membrane and then resulting in cochleotopic tuning. The results presented here emphasize that the tonotopic arrangement of the cochlea may limit the use of 500-Hz as a stimulus frequency for real-time ECochG monitoring during insertion. This notion is particularly important for patients with smaller cochleas and deeper insertions.

There were three different types of electrodes in this study, and all were approximately 20 mm in length. However, with the new slim lateral wall electrode (CI624) used in this study, a maximum of 358-degree apical insertion angle can be achieved although the average apical insertion angle being only 277.9 degrees. The CM amplitude peak for 500-Hz was only seen at a more basal electrode than the most apical for one insertion where the cochlear diameter was 8.4 mm and the apical insertion angle was 358 degrees (i.e., in the smallest cochlea with the largest apical insertion angle for the lateral wall electrode). Thus, crossing CF is likely not a concern for most insertions when using this lateral wall electrode or those of similar length.

The variability of where different frequencies are identified along the cochlea based on cochlear size is not a new concept. In fact, Greenwood’s frequency position function (Greenwood, 1990), the mathematical basis for CI electrode programming, takes into account the cochlear duct length (directly related to cochlear size) to determine the precise location where the CF should be present. For fixed electrode lengths with variable cochlear sizes, the CF for a fixed frequency (e.g., 500-Hz) is expected to be localized at a shorter distance along the cochlear duct length for smaller cochleas and at a longer distance along the cochlear duct for larger cochleas (Avci et al., 2014; Pietsch et al., 2017; Dhanasingh, 2018).

### Real-Time Multifrequency Electrocochleography as a Hearing Preservation Tool

No real-time feedback with multifrequency ECochG was used during the insertions for this study as the utility of multifrequency ECochG has previously not been described. A primary application of real-time feedback with ECochG is to guide the surgical decision-making process. The insertion of the final few millimeters of the electrode has the potential to impact whether hearing is preserved or lost, without having any direct impact on electric only performance. In patients with significant residual hearing, the surgeon’s decision to insert the full array as opposed to leaving electrode contacts outside of the cochlea may have lasting consequences. If there is a robust response at the end of full insertion, hearing preservation is likely, while a declining response may indicate that stopping insertion may be necessary to preserve hearing (Harris et al., 2017a,b; Lenarz et al., 2022). A complete loss of response at the end of insertion may indicate that hearing preservation is unlikely, and a full insertion is the best option (Harris et al., 2017a).

Here, we show that understanding these intraoperative ECochG responses may follow a different paradigm, especially if 500-Hz is used as the stimulus frequency. When using both 250- and 500-Hz acoustic stimulus, multifrequency ECochG allows the surgeon to understand whether a declining response is related to either crossing CF place or intracochlear trauma. If both the ECochG response to 250- and 500-Hz declines simultaneously (i.e., synchronous), there is likely intracochlear trauma and stopping insertion while partially withdrawing the electrode may allow for recovery of the ECochG response which may preserve hearing. If 500-Hz response declines while 250-Hz continues to rise (i.e., dyssynchronous), the electrode is likely passing the CF for 500-Hz and continued insertion is acceptable. If both 250- and 500-Hz response continue to rise at full insertion, the electrode has not likely passed CF for either frequency and hearing preservation is likely. If multifrequency ECochG is not available, it appears that monitoring 250-Hz will likely yield the best outcomes for hearing preservation as CM amplitude drops related to 250-Hz are more strongly correlated with LFPTA shift when compared to CM amplitude drops for 500-Hz (*r* = 0.58 vs. 0.31). For the lateral wall electrode used in this study, crossing the 500-Hz CF place was uncommon as most insertions have an apical insertion angle < 350 degrees. One would expect that if longer lateral wall arrays were used that achieved apical insertion angles beyond 350 degrees, CF for 500-Hz would again likely influence interpretation in a manner similar to the perimodiolar array findings in the present study. Future studies should confirm this generalization. Whether multifrequency ECochG as opposed to monitoring 250-Hz alone for insertion feedback results in better hearing preservation was not investigated in the current study. Prospective studies are currently underway comparing hearing preservation results when using a single stimulus frequency of 250-Hz and multifrequency ECochG using both 250- and 500-Hz.

### Limitations

While this study was able to use multifrequency ECochG to better understand ECochG as a hearing preservation tool, there are several notable limitations. In prior studies (O’Leary et al., 2020; Lenarz et al., 2022; Walia et al., 2022) including this study, there is significant variability related to hearing preservation that is only partially explained by ECochG drop for individual subjects. This is not surprising as insertions that may appear completely atraumatic from an ECochG standpoint may lose hearing through other postoperative mechanisms such as fibrosis or foreign body response (Quesnel et al., 2016). Conversely, not all drops in CM amplitude may be related to irreversible trauma as the electrode array may place pressure at a more basal portion of the cochlea early during insertion that may be relieved as the electrode is further advanced. Additionally, as shown in this study, the CM drops may be related to interactions with the CF location in the cochlea. The CM amplitude drop is only a rough approximation for intracochlear trauma that may affect hearing outcomes. Despite that, CM amplitude drops can be predictive of decline in residual hearing as shown in the current study and other recent studies (O’Leary et al., 2020; Bester et al., 2021; Lenarz et al., 2022). Importantly, CM activity is related to outer hair cell activity but not necessarily hearing ability; thus, there may be hair cells measured by the CM that are not innervated by functional spiral ganglion cells (Fontenot et al., 2019). This may explain some of the variability with changes in LFPTA and hearing preservation that is unexplained by CM amplitude drops. Future studies must also evaluate other ECochG signal components including changes in latency during signal decay and its impact on hearing preservation once active interpretation of these features are possible intraoperatively.

Another consideration is that real-time multifrequency ECochG was not used to make any adjustments during insertion in the current study. Thus, the ability for a surgeon to respond to multifrequency ECochG was not assessed. Future studies will have to assess whether the CM amplitude drops noted for multifrequency ECochG can be reversed to allow for an atraumatic electrode insertion. The feedback must be timely to provide the surgeon with an opportunity to pause, retract, and reposition the electrode so that further electrode advancement will not result in a continued drop in CM amplitude.

## Conclusion

Previous studies have exclusively focused on using 500-Hz as the acoustic stimulus during intraoperative, intracochlear real-time ECochG monitoring for the purposes of hearing preservation. Multifrequency ECochG is a newly available technology that can provide simultaneous real-time feedback from multiple acoustically evoked frequencies and the associated changes in CM amplitudes during CI surgery. The changes in CM amplitude during 250-Hz stimulus were more predictive of hearing preservation than the changes in CM amplitude for 500-Hz stimulus. This is likely due to the electrode array passing the CF for 500-Hz during insertion which may be misinterpreted as intracochlear trauma if 500-Hz acoustic stimulus is used, which is relevant for insertions where the cochlear diameter is <9.5 mm (i.e., smaller cochleas) and the apical insertion angle is > 350 degrees. This is particularly important for the perimodiolar electrode where apical insertion angles are often deeper than 350 degrees, as opposed to lateral wall electrode used in the present study (average apical insertion angle, 278 degrees). Multifrequency ECochG allows the surgeon to differentiate between intracochlear trauma and advancement of the apical electrode beyond the CF. Thus, a synchronous drop in both frequencies during insertion is likely related to basilar membrane impact, while a rise in 250-Hz and drop in 500-Hz is representative of passing the CF.

## Data Availability Statement

The raw data supporting the conclusions of this article will be made available by the authors, without undue reservation.

## Ethics Statement

The studies involving human participants were reviewed and approved by Washington University in St. Louis Institutional Review Board (#202007087). The patients/participants provided their written informed consent to participate in this study.

## Author Contributions

AW and CB contributed to the study concept, organization, and manuscript inception. AW, CB, MS, and AO contributed to the research design. AW, MS, SL, CW, TH, ND, JH, and CB contributed to data collection. AW, SL, and DK contributed to data analysis and tools. AW, CB, CW, DK, TH, and AO contributed to the draft revision. All authors contributed to the article and approved the submitted version.

## Conflict of Interest

CW was a consultant for Stryker and Cochlear Ltd. JH was a consultant for Cochlear Ltd. CB was a consultant for Advanced Bionics, Cochlear Ltd., Envoy, and iotaMotion and has equity interest in Advanced Cochlear Diagnostics, LLC. The remaining authors declare that the research was conducted in the absence of any commercial or financial relationships that could be construed as a potential conflict of interest.

## Publisher’s Note

All claims expressed in this article are solely those of the authors and do not necessarily represent those of their affiliated organizations, or those of the publisher, the editors and the reviewers. Any product that may be evaluated in this article, or claim that may be made by its manufacturer, is not guaranteed or endorsed by the publisher.
